# Recent Advances in the Use of Metal-Organic Frameworks for Dye Adsorption

**DOI:** 10.3389/fchem.2020.00708

**Published:** 2020-08-28

**Authors:** Vonika Ka-Man Au

**Affiliations:** Department of Science and Environmental Studies, The Education University of Hong Kong, Hong Kong, China

**Keywords:** metal-organic frameworks, adsorption, dyes, porous materials, remediation

## Abstract

Organic dyes are heavily used in industries for the manufacture of colored goods. This has eventually resulted in the generation of contaminated wastewater which is hard to be purified. Recent studies have demonstrated that metal-organic frameworks (MOFs), a class of supramolecular materials of immense interest, are useful in the adsorption of organic dye molecules because of their modifiable porous structures. In this mini review, the recent advances in the use of MOFs for the adsorption of organic dyes will be summarized.

## Introduction

Dyes are widely used in the production of everyday goods ranging from paper and textiles to food and pharmaceuticals. Consequently, there have been increasing concerns about the contamination of wastewater by dyes due to their highly visible colors and persistence. Physical, biological, and chemical methods have been developed for the removal of dye molecules from wastewater (Bhatia et al., 2017; Katheresan et al., 2018). However, these methods usually require the use of sophisticated systems and the costs of operation are high (Robinson et al., 2001). On the other hand, adsorption removal by metal-organic frameworks (MOFs) represents a relatively low-cost and efficient alternative (Khan et al., 2013). MOFs are a class of versatile porous materials that have been extensively studied over the last two decades (Long and Yaghi, 2009; Zhou and Kitagawa, 2014). With a vast selection of ligands and metal nodes, MOFs with different sizes and topologies can be constructed (Doonan et al., 2009; Cohen, 2010; Stock and Biswas, 2012; Lu et al., 2014). As the regular and directional framework structures of MOFs can be visualized with the help of X-ray diffraction studies, precise designs of MOFs with desired pore sizes and surface functional groups are possible, rendering them useful candidates for the adsorption of gases (Li et al., 2009; Fukushima et al., 2010) and many other applications (Janiak and Vieth, 2010; Furukawa et al., 2013; Hu et al., 2014; Lin et al., 2014). The miniaturization of MOFs into nanosheets (Au et al., 2019; Liu et al., 2019) and nanoparticles (Feng et al., 2019) has also broadened the potential applications of MOFs. Over the past decade, the effectiveness of MOFs for the adsorption of organic dye molecules in liquid systems have been recognized. A range of organic dye molecules, regardless of their chromophoric backbones, has been demonstrated to be adsorbable on MOFs (Ayati et al., 2016; Jiang et al., 2019). Herein, examples will be selected from the literature to provide an overview of the recent development of dye adsorption using MOF-based materials, starting from primitive and functionalized MOFs to MOF-based composites.

## MOFs for Dye Adsorption

The judicious selection of metal nodes and ligands has allowed the preparation of MOFs with different geometries, topologies, and pore sizes. In order to prepare porous frameworks for the selective adsorption of different organic dye molecules, a number of strategies such as functionalization of organic linkers, modulation of non-covalent interactions, and defect engineering have been developed, and these will be described below.

Electrostatic interactions are regarded as one of the most important types of non-covalent interactions involved in the binding of dye molecules by MOFs given the availability of a library of anionic and cationic dyes. These dyes have been extensively used in industries for dying cotton, silk, and wood (Rafatullah et al., 2010). Due to the toxicity and highly extensive usage of ionic dyes, a number of MOFs has been developed for their sensing and capture. In 2010, Jhung and co-workers reported the use of two MOFs based on chromium terephthalates, namely MIL-53(Cr) and MIL-101(Cr), for the adsorptive removal of methyl orange (Haque et al., 2010). Both MOFs were found to be superior to activated carbon toward the adsorption of methyl orange. MIL-101(Cr), which exhibited larger porosity than MIL-53(Cr), was observed to show a higher adsorption capacity. The group further modified MIL-101(Cr) by grafting with ethylenediamine to give ED-MIL-101(Cr) and subsequent protonation to give PED-MIL-101(Cr) (Haque et al., 2010). Despite the slightly reduced porosity and pore sizes, the grafted MOFs showed higher adsorption capacities in the order PED-MIL-101(Cr) > ED-MIL-101(Cr) > MIL-101(Cr). Since protonated PED-MIL-101(Cr) was positively-charged and ED-MIL-101(Cr) also exhibited a partial positive charge, it was suggested that electrostatic interactions were involved in the adsorption mechanism and hence the cationic PED-MIL-101(Cr) MOF showed the strongest interaction with the anionic methyl orange molecules.

The same group then extended their work to a MOF based on iron terephthalate, MOF-235(Fe) or [Fe_3_O(terephthalate)_3_(DMF)_3_][FeCl_4_] (Haque et al., 2011). This MOF was found to adsorb both anionic methyl orange and cationic methylene blue dyes in the liquid phase even though it was found to be non-porous to nitrogen at low temperature. Electrostatic interactions were suggested to be responsible for the adsorption properties of MOF-235(Fe) as in the case of MIL-101(Cr). As MOF-235(Fe) was composed of both cationic framework units and charge-balancing anions, the material was capable of simultaneously binding both methyl orange and methylene blue in aqueous solutions. In addition, the rate of the adsorption process was found to vary with pH. At higher pH, the density of the positive charge of MOF-235(Fe) would be lower and thus reduced adsorption of methyl orange would be observed. At low pH, the density of the negative charge of the MOF would increase, leading to increased adsorption of methylene blue.

In addition to the surface charges on MOFs, a good match in size and geometry between the MOF and the dye is another important factor governing the adsorption process. In 2010, Lin and co-workers prepared a series of three-dimensional 4,4-connected MOFs based on copper paddle-wheel secondary building units and tetracarboxylate linkers derived from tetraphenylmethane (Liu et al., 2010). According to nitrogen and hydrogen adsorption studies at 77 K, the MOFs exhibited permanent porosities and were thus used for inclusion studies with the dye Brilliant Blue R-250 (BBR-250). The dye uptake of the 4,4-connected MOFs were found to strongly correlate to their pore sizes. MOFs with pore sizes smaller than the dimensions of BBR-250 were observed to show minimal dye uptake due to surface adsorption, whereas a maximum dye uptake capacity of 73 wt % was demonstrated by the MOF with largest pore size.

**Figure 1:**
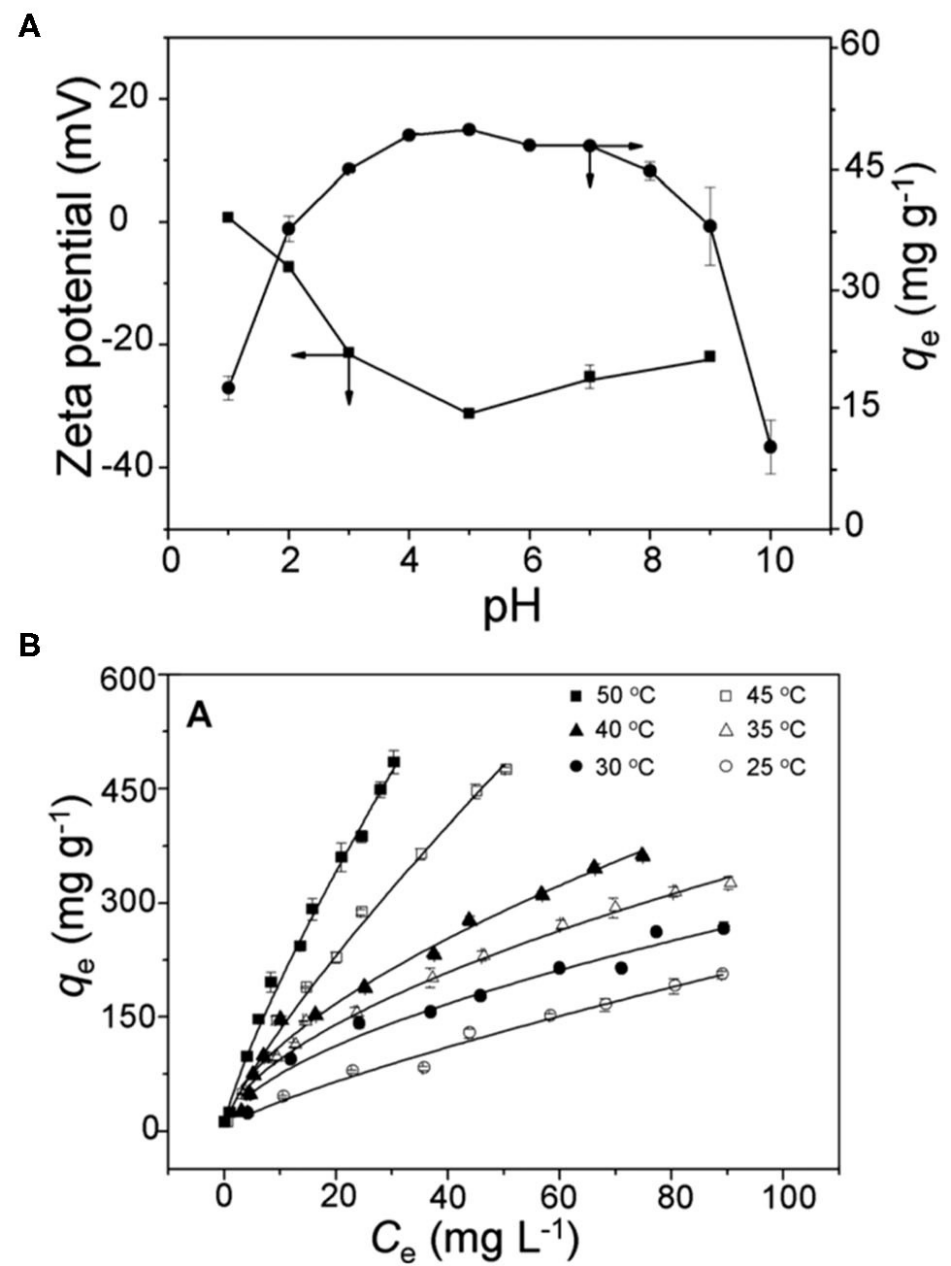
**(A)** Effect of pH on the adsorption of malachite green (100 mg L^−1^) on MIL-100(Fe) (10.0 mg) containing 0.01 M NaCl, and the zeta potential of MIL-100(Fe) (0.4 mg L^−1^) at 30°C; **(B)** adsorption isotherms for malachite green on MIL-100(Fe) at different temperatures and pH 5.0. Reproduced from Huo and Yan (2012) with permission from the Royal Society of Chemistry.

Using trimesic acid as the organic linker, Yan and co-worker prepared MIL-100(Fe) for the adsorption of the cationic triphenylmethane dye, malachite green (Huo and Yan, 2012). It was found the MIL-100(Fe) exhibited an experimental adsorption capacity of 205 mg g^−1^ at room temperature, which was much higher than those of MIL-101(Cr) and MIL-53(Al). The pH-dependence of the adsorption process and zeta potential measurements indicated that electrostatic interactions were involved in the adsorption mechanism (Figure 1A). In addition, π-π interactions also existed between the benzene rings in malachite green and MIL-100(Fe). The adsorption of malachite green on MIL-100(Fe) was shown to be endothermic, with an increase in the adsorption capacity at higher adsorption temperatures (Figure 1B). Thermodynamic studies further showed that for spontaneous adsorption of malachite green on MIL-100(Fe), a positive entropy change (ΔS) is favorable while a positive enthalpy change (ΔH) is unfavorable (Huo and Yan, 2012). This could be explained by the fact that water molecules occupied the open metal sites in MIL-100(Fe). Upon the interaction between the Lewis acidic Fe sites and the Lewis basic –N(CH_3_)_2_ groups in malachite green in aqueous solution, the coordinated water molecules would be replaced and released, giving rise to an increase in the entropy of the system. On the other hand, the lack of open metal sites in MIL-53(Al) and the repulsion caused by surface positive charges on MIL-101(Cr) would lead to poorer adsorption of malachite green.

On the other hand, functional groups such as amino substituents could be introduced to the linkers to enhance the non-covalent interactions with organic dye molecules to improve the adsorption capacity (Wang and Cohen, 2009; Tanabe and Cohen, 2010). Church and co-workers functionalize MIL-101(Al) with amino groups to obtain NH_2_-MIL-101(Al) with a high adsorption capacity for methylene blue of up to 762 mg g^−1^ (Haque et al., 2014). The presence of the amino moieties on the MOF surface has led to an increase in the electrostatic interactions between the MOF and methylene blue. However, the X-ray photoelectron spectrum of the MOF revealed that disruptions in the MOF structure occurred during the adsorption process and approximately 30% of the Al(III) ions were lost to the solution, making the MOF non-reusable. On the contrary, NH_2_-MIL-101(Al) remained intact and could be recycled upon the adsorption of anionic methyl orange. In another work, Xiong and co-workers utilized the amino function group to prepare NH_2_-MIL-53(Al) for the adsorption of the cationic dyes, methylene blue, and malachite green (Li et al., 2015). Comparison of the pH-dependent adsorption studies of NH_2_-MIL-53(Al) and its unsubstituted analog, MIL-53(Al), showed that the adsorption process was not driven by electrostatic interactions in this case. Instead, strong hydrogen bonding interactions were observed between the amino hydrogens of NH_2_-MIL-53(Al) and the nitrogen atoms on methylene blue or malachite green. Weak π-π interactions also existed between the benzene rings of NH_2_-MIL-53(Al) and the dye molecules. Similar modifications of other MOFs by the amino group have also led to the enhanced adsorption of different organic dyes (Chen et al., 2015; Fan et al., 2018).

Using organic linkers with different structural backbones, MOFs with different topologies and cavity sizes can be rationally constructed (Lyu et al., 2019). For instance, the tetratopic carboxylate ligand, namely 3,3′,5,5′-tetrakis(*p*-carboxyphenyl)-2,2′,6,6′-tetramethoxy-1,10-biphenyl, was used to prepare an anionic trinuclear cadmium MOF (Seth et al., 2015). This cadmium MOF exhibited high flexibility and robustness, and would allow postsynthetic metal exchange with a number of main group and lanthanide ions to give new MOFs with the same structure. In particular, cationic MOFs would be produced when the divalent cadmium ions were exchanged by trivalent lanthanide ions, leading to MOFs with essentially different dye adsorption properties. It was observed that the parent cadmium MOF preferentially adsorbed cationic methylene blue from a mixture containing a neutral or an anionic dye; whereas the isostructural europium MOF could selectively adsorb the anionic dye, bromophenol blue, from a mixture with either a cationic or a neutral dye.

**Figure 2:**
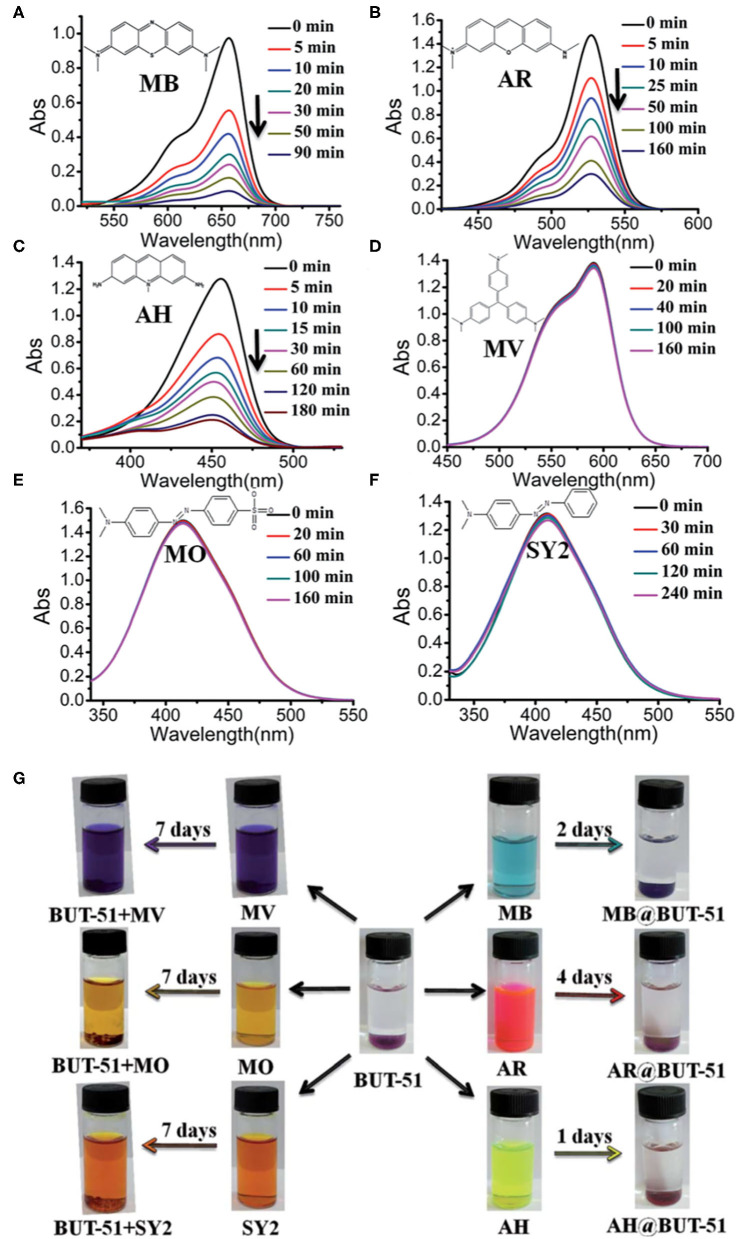
UV-Vis spectra of acetone solutions of **(A)** methylene blue (MB), **(B)** acridine red (AR), **(C)** acriflavine hydrochloride (AH), **(D)** methylene violet (MV), **(E)** methyl orange (MO), and **(F)** solvent yellow 2 (SY2) in the presence of BUT-51 monitored with time and **(G)** the respective photographs before and after dye adsorption. Reproduced from Han et al. (2015) with permission from the Royal Society of Chemistry.

In 2015, Li and co-workers reported the nanotubular anionic MOF, {[(CH_3_)_2_NH_2_][Co_2_NaL_2_(CH_3_COO)_2_]·*x*S}_n_ (BUT-51; S = solvent) using 5-(pyridine-4-yl)isophthalic acid (H_2_L) as the linker (Han et al., 2015). The cobalt MOF featured two types of nanotubular channels with pore diameters of 14 and 6.5 Å, respectively. This anionic framework was found to be porous to cationic dyes including methylene blue, acridine red, and acriflavine hydrochloride, but non-porous to the larger methylene violet dye, neutral dyes such as solvent yellow 2, as well as anionic dyes such as methyl orange (Figure 2). In addition to electrostatic interactions, the adsorption properties of the cobalt MOF could be attributed to size- and shape-exclusive effects. It was suggested that cationic dye molecules would be adsorbed via the larger channel of the MOF via a cation exchange mechanism, in which the cationic [(CH_3_)_2_NH_2_]^+^ counterion would be released from the MOF through the smaller nanotubular channel. Cationic dyes with larger sizes such as methyl violet molecules could not enter the nanotubular channels of the MOF due to a mismatch in size and shape, and therefore could not be adsorbed. On the other hand, reversibility tests demonstrated that acriflavine hydrochloride was preferentially adsorbed and could hardly be released from the MOF due to the strong coordination between the amino group of the dye molecule and the unsaturated Co(II) ion. More recently, a single-walled metal-organic nanotube with an armchair (3,3) topology, ([CH_3_NH_3_][Zn(NTB)(NMF)]·4.5NMF (H_3_NTB = 4,4′,4″-nitrilotrisbenzoic acid, NMF = *N*-methylformamide), has been reported to exhibit an interior channel diameter of 21 Å (Zhou et al., 2018). The large open mesoporous channels have allowed the metal-organic nanotube to adsorb common dye molecules with high efficiencies. In particular, adsorption capacities of over 1650 mg g^−1^ were reached for the carcinogenic dyes basic red 9 and basic violet 14.

A further approach to the modification of MOFs is defect engineering (Wu et al., 2013; Fang et al., 2015). Defects could be generated in MOFs via the use of modulator compounds such as acetic acid and benzoic acid, postsynthetic treatment with acids, or the mixed-linker approach (Jiang et al., 2019). Very often, defective MOFs were found to exhibit higher porosities and surface areas and thus enhanced adsorption properties because of the missing linkers. In 2016, Huang and co-workers reported the synthesis of defective UiO-66(Zr) using benzoic acid as a modulator and hydrochloric acid for postsynthetic treatment (Wang et al., 2016). The defective MOF was found to show over 9 times higher adsorption capacity of safranine T (366 mg g^−1^) than its defect-free analog (30 mg g^−1^). Despite the larger cavity size, the defective UiO-66(Zr) displayed a relatively uniform distribution of pore sizes and was capable to selectively adsorb safranine T over crystal violet due to a size-exclusion effect. In a related study, cetyltrimethylammonium bromide (CTAB) was used as a modifier to induce more missing-linker defects in UiO-66 and resulted in a higher adsorption capacity of the MOF (Zhang et al., 2019).

## MOF-Based Composite Materials for Dye Adsorption

In recent years, MOFs have been engineered in many ways to fine-tune their properties. One simple method is to make use of host-guest chemistry and modify the properties of the MOFs with the dye (guest) molecules (Wuttke et al., 2014). As dyes are intensely colored and some also exhibit strong luminescence (Allendorf et al., 2009; Cui et al., 2012), there has recently been increasing attention toward the use of MOFs encapsulated with dyes (dye@MOFs) as new classes of functional materials. Wu and co-workers used (*E*)-4-(2-carboxyvinyl)benzoic acid (H_2_L) to prepare the cadmium MOF, [CdL(H_2_O)]·4DMF·2H_2_O (Dong et al., 2014). The MOF was found to adsorb rhodamine B (RhB) to form a luminescent adduct. While the emission of RhB was quenched in a ground mixture with the MOF, ligand-to-dye energy transfer occurred in RhB@MOF such that both the ligand and RhB emissions were observed at 420 and 595 nm, respectively. The relative intensity of the RhB emission would increase with a higher dye loading, and thus tunable emission was achieved. Upon exposure to different volatile organic compounds (VOCs), RhB@MOF was found to exhibit guest-dependent emission peak-height ratios of the ligand L to RhB. Interestingly, RhB@MOF could also be used to differentiate molecules with very similar structures such as *o*-, *m*- and *p*-xylenes, as well as fluoro-, chloro-, and bromobenzenes, rendering it a useful luminescent probe for the sensing of VOCs. A supramolecular cadmium framework was also prepared using cucurbit[10]uril (CB[10]) as the organic linker (Yao et al., 2017). Upon the encapsulation of rhodamine B, pyrenemethanamine hydrochloride and bathocuproine hydrochloride, the framework was observed to exhibit red-green-blue fluorescence, respectively.

An alternative approach is to combine MOFs with another type of nanomaterial to create composite materials that exhibit synergistic properties from both materials (Zhu and Xu, 2014; Li and Huo, 2015). This method is especially useful for the enhancement of the robustness, stability, and applicability of MOFs for their utilization in practical scenarios. Because of its high adsorption capacity and strong dispersive forces within its densely packed layer structure, graphene and graphite oxide (GO) derivatives represent one of the most common types of nanomaterials for composite formation with MOFs (Petit and Bandosz, 2009). As early as in 2010, composites of GO and MOFs such as MOF-5 and HKUST-1 have been prepared by the group of Bandosz for the adsorption of ammonia (Petit and Bandosz, 2010; Petit et al., 2010).

Recently, the formation of MOF/GO composites has been demonstrated to increase the thermal stability of the porous material, giving rise to enhanced adsorption of dyes (Ma et al., 2017). For example, the composites from MIL-100(Fe) and graphene oxide nanosheets were found to exhibit a sandwich-like structure with the decomposition temperature increased from 280 to 350°C (Luo and Wang, 2018). This composite material was found to exhibit enhanced adsorption capacities of methyl orange and methylene blue from aqueous solution at a 5 wt% loading of graphene oxide. MOF composites have also been prepared from ZIF-8 with graphene oxide or carbon nanotubes (Abdi et al., 2017). In this case, the composite material exhibited much enhanced adsorption capacities for malachite green, with maximum values of 1,667, 2,034, and 3,300 mg g^−1^ for ZIF-8, ZIF-8@CNT, and ZIF-8@GO, respectively, at ambient temperature. The composite materials were found to show higher uptake of malachite green at elevated temperatures, and retained its adsorption ability in real wastewater, indicating their potentials for practical applications (Abdi et al., 2017).

More sophisticated composite materials have also been obtained from the combination of HKUST-1, graphene oxide nanosheets, and magnetic Fe_3_O_4_ nanoparticles (Li et al., 2014). The resulting Fe_3_O_4_/HKUST-1/GO hybrid was magnetic in nature. The adsorption properties of the composite material toward methylene blue was investigated. Despite a reduced specific surface area, the three-component Fe_3_O_4_/HKUST-1/GO exhibited a good match in channel diameter and molecular width with methylene blue, and therefore showed a higher dye adsorption capacity than the simpler Fe_3_O_4_/HKUST-1 composite. In addition to physical adsorption, irreversible chemical adsorption via the coordination of nitrogen atoms in methylene blue with the copper atoms in HKUST-1 also played a minor role in the adsorption mechanism. From desorption and regeneration experiments, the adsorption capacity of Fe_3_O_4_/HKUST-1 was found to decline rapidly with increasing numbers of cycles, from nearly quantitative adsorption to only 60% after recycling for 5 times. In the presence of graphene oxide as a constituent material, only slight reduction in the adsorption capacity of methylene blue was observed for Fe_3_O_4_/HKUST-1/GO and the value remained over 90% after 5 cycles (Li et al., 2014).

Ultrasmall nanoparticles of various MOFs, such as HKUST-1, ZIF-8, and ZIF-67, supported on copper silicate nanotubes (CuSiNT), have also been prepared by either using the CuSiNT directly as a starting material or by doping other transition metal ions with CuSiNT (Qin and Zeng, 2018). The CuSiNT-supported HKUST-1 nanocomposites were demonstrated to show enhanced adsorption of methyl orange, congo red, thymol blue than their microsized couterparts. The improved adsorption capacity was attributed to the CuSiNT support, which could provide more open metal sites and accessible functional groups, and thus the diffusion barrier of the dye molecules could be reduced.

Other than inorganic nanomaterials, MOFs have also been modified with biomaterials to prepare hybrid composites. Sicard, Ricoux and co-workers immobilized the enzyme, microperoxidase-8, into nanoparticles of MIL-101(Cr) to form a matrix (Gkaniatsou et al., 2018). While the enzyme gained resistance to acidic or oxidative conditions under the protection by the extended network structure of MIL-101(Cr), it could also enhance the selectivity of the MOF toward methyl orange for oxidative degradation. Very recently, the groups of Lai, Zhou, and Zhan used three-dimensional (3D) printing technique to prepare MOF-polymer composites from HKUST-1 and a biocompatible binder made of calcium alginate and gelatin (Pei et al., 2020). It was observed that the introduction of the biocompatible blend would improve the mechanical properties of the 3D-printed material in water, probably due to the increased toughness of calcium alginate fibers upon swelling and hydrogel formation in water. The 3D-printed composites were found to adsorb a range of organic dyes including methylene blue, malachite green, methyl violet, rhodamine B, and auramine O, with the adsorption performance dependent on the printing geometry, as well as the size and loading of the MOF.

## Conclusion

Recent advances in the field of MOFs have led to the rapid development of novel adsorbents for organic dye molecules. From the functionalization of organic linkers to defect engineering and the fabrication of composite materials, the porous properties of MOFs could be readily modified to adapt for the adsorption of cationic, anionic, and non-ionic dyes. With further improvement in the adsorption capacity, stability, and regenerability, it is anticipated that MOFs will show great potential for practical applications in wastewater treatment.

## Author Contributions

The author confirms being the sole contributor of this work and has approved it for publication.

## Conflict of Interest

The author declares that the research was conducted in the absence of any commercial or financial relationships that could be construed as a potential conflict of interest.
